# Early detection of canine hemangiosarcoma via cfDNA fragmentation and copy number alterations in liquid biopsies using machine learning

**DOI:** 10.3389/fvets.2024.1489402

**Published:** 2025-01-13

**Authors:** Soohyun Ko, Jinhee Jang, Sun Shin Yi, ChangHyuk Kwon

**Affiliations:** ^1^GenesisEgo, Seoul, Republic of Korea; ^2^Department of Biomedical Laboratory Science, Soonchunhyang University, Asan, Republic of Korea; ^3^BK21 Four Project, Department of Medical Sciences, Soonchunhyang University, Asan, Republic of Korea

**Keywords:** canine, cell-free DNA, copy number alteration, fragment, hemangiosarcoma, liquid biopsies, machine learning

## Abstract

Hemangiosarcoma is a highly malignant tumor commonly affecting canines, originating from endothelial cells that line blood vessels, underscoring the importance of early detection. This canine cancer is analogous to human angiosarcoma, and the development of liquid biopsies leveraging cell-free DNA (cfDNA) represents a promising step forward in early cancer diagnosis. In this study, we utilized Whole Genome Sequencing (WGS) to analyze fragment sizes and copy number alterations (CNAs) in cfDNA from 21 hemangiosarcoma-affected and 36 healthy dogs, aiming to enhance early cancer detection accuracy through machine learning models. Our findings reveal that similar to trends in human oncology, hemangiosarcoma samples exhibited shorter DNA fragment sizes compared to healthy controls, with a notable leftward shift in the primary peak. Interestingly, canine hemangiosarcoma DNA fragment sizes demonstrated eight distinct periodic patterns diverging from those typically observed in human angiosarcoma. Additionally, we identified seven novel genomic gains and nine losses in the hemangiosarcoma samples. Applying machine learning to the cfDNA fragment size distribution, we achieved an impressive average Area Under the Curve (AUC) of 0.93 in 10-fold cross-validation, underscoring the potential of this approach for precise early-stage cancer classification. This study confirms distinctive cfDNA fragment size and CNA patterns in hemangiosarcoma-affected vs. healthy dogs and demonstrates the promise of these biomarkers in canine cancer screening, early detection, and monitoring via liquid biopsies. These findings establish a foundation for broader research on cfDNA analysis in various canine cancers, integrating methodologies from human oncology to enhance early detection and diagnostic precision in veterinary medicine.

## 1 Introduction

Canines are inherently susceptible to a diverse array of tumors, which pose a significant mortality threat. Tumor diagnosis in dogs is traditionally confirmed via tissue biopsy, with subsequent treatment often involving surgical excision of the tumor and pharmacological therapy (1). Among the numerous types of canine tumors, hemangiosarcoma is particularly malignant, originating from the endothelial cells that line the vasculature and manifesting in multiple organs, including the liver, spleen, and skin (2, 3). Its human analog, angiosarcoma, mirrors hemangiosarcoma in histological structure, molecular pathogenesis, and clinical presentation, thereby facilitating comparative oncological studies between species. Recent advances in the study of cell-free DNA (cfDNA) have highlighted its potential for non-invasive, early detection and monitoring of human cancers (4–10).

As a byproduct of cancer tissue degradation, cfDNA circulates in the bloodstream, with its baseline levels detectable in healthy individuals. Alterations in plasma cell-free DNA (cfDNA) concentrations, modifications in cfDNA methylation patterns, mutations originating from cancer cells, and the phenomenon of loss of heterozygosity emerge as promising biomarkers for the diagnosis of cancer (11, 12).

Incorporating machine learning with cfDNA analysis has paved a new approach for advanced cancer diagnosis (13–16), leveraging genomic insights from Whole Genome Sequencing (WGS) data, such as copy number alterations (CNA), fragment size distribution, and genomic breakpoints. While the exploration of cfDNA liquid biopsies in canines is less developed compared to human studies, emerging research hints at its potential.

Notably, investigations by Favaro et al. have delineated the enhanced detection of circulating tumor DNA (ctDNA) in malignant vs. benign lesions in canine splenic hemangiosarcoma, highlighting its utility in the diagnosis, monitoring, and detection of this specific tumor type. A pioneering aspect of cancer screening is the analysis of cfDNA fragment patterns. Sequencing data reveal discernible discrepancies in fragment size distribution between normal and cancerous samples, characterized by an enrichment of both longer and shorter fragments in cancer specimens (14, 17–20).

Furthermore, the periodicity of peaks and troughs at 10 bp intervals offers additional discriminative features for distinguishing between malignant and benign samples (14). Our methodology proposes a novel screening paradigm by analyzing cfDNA fragment size distribution in conjunction with machine learning techniques. We employ an array of machine learning algorithms to classify hemangiosarcoma from normal samples, focusing on the distribution of fragment sizes within specific ranges and delving into the realm of copy number alterations (CNA), thus contributing to the expanding frontier of veterinary oncological research.

## 2 Materials and methods

### 2.1 Overview and data

**Figure 1:**
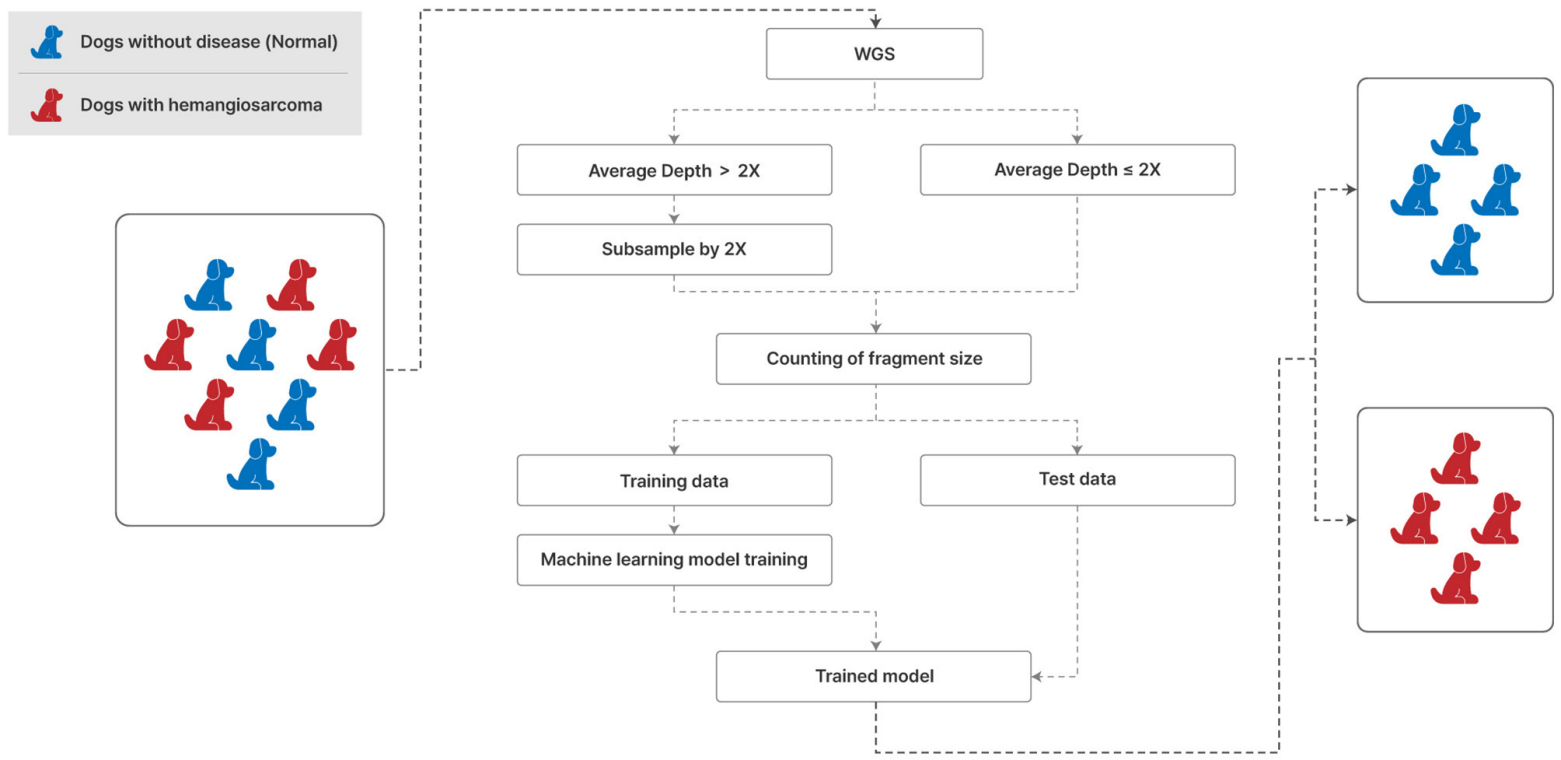
Analysis and processing procedures of WGS from liquid specimens of the normal and hemangiosarcoma groups. 2× depth (mean coverage) means that approximately twice as much data as the canine genome (genome size ≈ 2.4 Gb) is produced and analyzed. We used 70% of the data for the training set and 30% for the test set and applied seven classification models.

This study unfolds as illustrated in Figure 1, commencing with the alignment of Whole Genome Sequencing (WGS) data to the canFam3.1 (21) reference genome via Bwa-mem version 0.7.17 (22), followed by the elimination of PCR duplicates with Picard version 1.81. Utilizing Samtools version 1.7 (23), we sorted the aligned data and computed the average sequencing depth. To maintain uniformity, samples exhibiting an average depth exceeding 2X were downscaled to a maximum depth of 2X. This subsampled dataset was then employed to enumerate fragment sizes using Samtools, laying the groundwork for feature selection to discern significant fragment dimensions. Subsequently, machine learning algorithms were engaged to distinguish hemangiosarcoma from non-cancerous samples. The dataset encompasses 20 diverse dog breeds, incorporating 21 hemangiosarcoma-afflicted dogs and 9 healthy controls from the PRJNA823593 Bio Project, in addition to 27 normal samples collated by our team. Of the 57 samples (Supplementary Tables 1, 2), 30 were analyzed using WGS data from Favaro et al. [(17), PRJNA823593], and WGS was performed on 27 samples confirmed as normal through CRP, serum chemistry, and CBC tests.

### 2.2 NGS protocol for cfDNA extraction

Normal group blood samples were obtained from professional breeders in South Korea, utilizing Roche CE-IVD cell-free DNA collection tubes (#07785666001) for blood collection. A two-stage centrifugation process was executed to isolate plasma, which was then preserved at −80°C for subsequent cfDNA extraction.

The MagMAX cell-free DNA Isolation Kit by Thermo Fisher Scientific Inc. was employed for plasma cfDNA extraction, adhering to the manufacturer's guidelines. The NEBNext Ultra II DNA Library Prep Kit for Illumina facilitated NGS library construction from the extracted cfDNA, with sequencing indices procured from NEBNext Multiplex Oligos for Illumina (Dual Index). An initial cfDNA input of 2ng was specified, with the full amount used for samples below this threshold. The library was prepared without physical or enzymatic fragmentation, following a size selection-free protocol per the manufacturer's directions. The Qubit™ dsDNA HS Assay Kit and the Qubit™ 4 Fluorometer were utilized for quantitative assessments, with the Agilent Cell-free DNA ScreenTape & reagents and DNA ScreenTape & reagents alongside the Tapestation 4150 deployed for quality evaluations. Sequencing was conducted on the Illumina NovaSeq 6000, adopting a 150PE approach for WGS.

### 2.3 Fragment size distribution analysis

**Figure 2:**
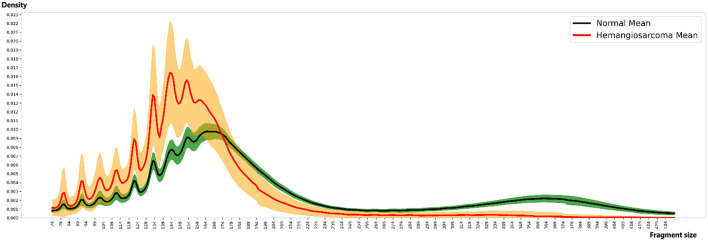
Fragment size distribution. The distinct patterns of fragment size distribution for hemangiosarcoma and normal samples, with means and variations represented by colored lines and areas. Fragment sizes were calculated in 5 bp increments, ranging from 74 to 434 bp. The *X*-axis represents fragment size, while the *Y*-axis indicates the proportion (density) of each size. In the graph, the red line represents the mean fragment size of hemangiosarcoma samples, with the orange color indicating the standard deviation. Similarly, the black line represents the mean fragment size of normal samples, and the green color denotes the standard deviation. The fragment pattern of the green-colored composition sample moves as a cohesive group. In contrast, the fragment pattern group of the orange-colored hemangiosarcoma sample is separated to create a more dramatic effect. Notable differences were observed, particularly within the 167 bp range, with the two groups showing the most significant similarity at 124, 137, and 147 bp.

The analysis of fragment size distribution employed samtools on aligned BAM files to tally fragment sizes ranging between 74 and 439 bp across all samples, subsequently calculating the distribution. For fragment size counting, samtools was configured with flag 66 and quality 30. Considering sample depth variability, scaling adjustments were made so the aggregate fragment size count equaled 1 for each sample. This normalized distribution was illustrated in Figure 2, utilizing Python version 3.6.13 and Matplotlib version 3.3.4.

### 2.4 Copy number alteration

We applied ichorCNA version 0.3.2 and WisecondorX (available at https://github.com/CenterForMedicalGeneticsGhent/WisecondorX.git) (24, 25) for CNA assessment, utilizing a 500 kb bin size for CNA computations. A normative panel for ichorCNA and a reference for WisecondorX were constituted using 27 normal samples. ichorCNA analysis included the generation of Mappability and GC content (26) for canFam3.1 with a 500 kb bin size via HMMcopy. The convergence of gain and loss regions across the ichorCNA and WisecondorX outcomes was facilitated by bedtools, identifying common regions of gain and loss between both methodologies.

### 2.5 Machine learning application

Machine learning was employed for feature selection and classification within the fragment size distribution dataset, distinguishing between Normal and Hemangiosarcoma samples via Python's scikit-learn version 0.24.2. Feature selection leveraged the support vector machine (SVM) (27) with recursive feature elimination (RFE) (28) executed iteratively 100 times to pinpoint optimal feature positions. This analysis was paralleled with a comparative study of oscillation peak and valley positions within the fragment size distribution against human counterparts.

For classification, the study utilized a cohort of 57 samples, split into a 70% training set and a 30% test set, with machine learning models including XGBoost (29), Random Forest (30), SVM, Extra Trees (ETC) (31), Gradient Boosting (GBC) (32), AdaBoost (33), and Bagging (BC) (34) from scikit-learn. Performance metrics like AUC score, accuracy, sensitivity, and specificity were calculated, iterating the process 10 times to ensure reliability.

## 3 Results

### 3.1 Fragment size distribution between normal and hemangiosarcoma

We analyzed the distribution of fragment sizes for both healthy samples and those afflicted by hemangiosarcoma. Our analysis comprised 36 normal samples and 21 hemangiosarcoma samples, from which we determined the mean fragment sizes and their standard deviations. These statistics are visually represented in Figure 2: the hemangiosarcoma samples are depicted with a red line (mean) and orange shading (variation), whereas the normal samples are illustrated with a black line (mean) and green shading (variation). This graphical representation underscores a distinct divergence in fragment size distribution patterns between the two groups. Our findings revealed that the principal peak of fragment size for normal samples is located at 165 bp, while for hemangiosarcoma samples, it shifts 5 bp to the left, settling at 160 bp. This shift mirrors patterns observed in human cell-free DNA (cfDNA) studies, suggesting a biological consistency across species. Notably, hemangiosarcoma samples exhibited a higher proportion of fragments under 174 bp, whereas normal samples predominated in fragment sizes above this threshold. Both normal and hemangiosarcoma samples displayed oscillatory patterns to the left of the main peak, each with eight oscillation cycles. Table 1 lists the peak positions within these oscillations for normal samples, revealing that while most peak positions align between normal and hemangiosarcoma samples, notable deviations occur in the peaks and valleys of the 7th and 8th oscillations by ~1 bp.

**Table 1:** Comparison of peak positions: normal vs. hemangiosarcoma samples.

		**1 (81 bp)**	**2 (91 bp)**	**3 (102 bp)**	**4 (112 bp)**	**5 (122 bp)**	**6 (133 bp)**	**7 (144 bp)**	**8 (154 bp)**
Normal	Peak	0.0015	0.0021	0.0023	0.0028	0.0042	0.0065	0.0078	0.0091
	Valley	0.0010	0.0014	0.0018	0.0022	0.0029	0.0048	0.0071	0.0086
	Diff	0.0005	0.0006	0.0005	0.0006	0.0013	0.0017	0.0007	0.0005
Hemangiosarcoma	Peak	0.0029	0.0042	0.0046	0.0054	0.0089	0.0139	0.0164	0.0156
	Valley	0.0014	0.0021	0.0031	0.0040	0.0053	0.0091	0.0129	0.0130
	Diff	0.0015	0.0020	0.0015	0.0014	0.0036	0.0048	0.0035	0.0026

All peak positions have a more significant portion in hemangiosarcoma than in normal. From the 1st to the 4th peaks, the average values are 0.0022 for normal and 0.0042 for hemangiosarcoma, showing a near 2-fold increase (0.0020 difference). From the 5th to the 8th peaks, the averages are 0.0069 for normal and 0.0137 for hemangiosarcoma, with a near 2-fold increase (0.0068 difference). While the difference increases gradually from the 1st to the 4th peak, it becomes more pronounced and more than doubles from the 5th to the 8th peak. Moreover, the disparity between the peaks and valleys (diff = peaks – valleys) accentuates this distinction. In normal, the 5th and 6th show more than a 1.5-fold difference, and in hemangiosarcoma, the 5th, 6th, and 7th show more than a 1.3-fold difference compared to the average. Commonly, the 5th and 6th exhibit the largest difference between peaks and valleys. The greatest difference between the two groups' diffs is observed in the 6th, and the least difference is in the 4th.

Thus, our analysis revealed that hemangiosarcoma samples exhibit notable disparities in the peaks and valleys within each oscillation cycle when compared to normal samples. Specifically, the fragment pattern characteristic of hemangiosarcoma includes a predominance of shorter fragments and a more pronounced variance between the peaks and valleys. Furthermore, the principal peak in hemangiosarcoma samples is discernibly shifted to the left, relative to that of normal samples, underscoring a unique fragment distribution pattern that distinguishes hemangiosarcoma from normal tissue samples. These findings highlight the potential of fragment size analysis as a biomarker for differentiating between hemangiosarcoma and healthy states, thereby contributing to the early detection and diagnosis of this aggressive cancer in canines.

### 3.2 Copy number alteration analysis

In our study, we analyzed changes in the DNA of 21 dogs with hemangiosarcoma, focusing on which parts of the DNA had more (gains) or fewer (losses) copies than usual. We used two special software tools, ichorCNA and WisecondorX, to do this, setting both to look at sections of DNA that were 500 kb long. Each dog's DNA showed different areas where these gains and losses happened, and they covered large parts of the chromosomes. To get a clearer picture of common changes, we only looked at changes that showed up in more than 6 of the samples. Using ichorCNA, we found 24 areas with gains and 14 areas with losses. WisecondorX found nine areas with gains and 14 with losses. By comparing the results from both tools, we identified the chromosomes that commonly had gains—these were chromosomes 5, 6, 13, 14, 16, 20, 24, and 31. For losses, the chromosomes were 2, 10, 11, 14, 16, 27, 30, 33, 34, and 36. We also compared our findings with what other researchers have discovered, which is detailed in Table 2 of our paper, and you can find the specific areas of the chromosomes that changed in Supplementary Table 3. This approach helped us understand which DNA changes are common in dogs with hemangiosarcoma.

**Table 2:** Gains and losses through CNA analysis.

	**Gain**	**Loss**
Our	5, 6, 13, 14, 16, 20, 24, 31	2, 10, 11, 14, 16, 27, 30, 33, 34, 36
Thomas et al. (35)	13, 24, 31	16
Kennedy et al. (36)		16, 31

Our study's findings on changes in DNA copy numbers (CNA) in dogs with hemangiosarcoma support and build upon previous research. Consistent with the work of Thomas R., we discovered that certain chromosomes (13, 24, and 31) often exhibited DNA copy number gains, while chromosome 16 frequently showed losses. This finding aligns with Kennedy K.'s observations regarding losses in chromosome 16. However, our study also identified a gain in chromosome 31, which contrasts with some earlier studies.

Specifically, we observed a loss in the CDKN2A/B gene on chromosome 16, a gene previously linked to hemangiosarcoma in dogs (37). This loss was anticipated based on prior research. Additionally, genes such as VEGFA on chromosome 12, and KDR and KIT on chromosome 13, are frequently amplified in these cancers. Our study corroborated these gains and also identified an increase in the MYC gene, which is less commonly reported.

### 3.3 Machine learning for identifying cancer-specific patterns

**Figure 3:**
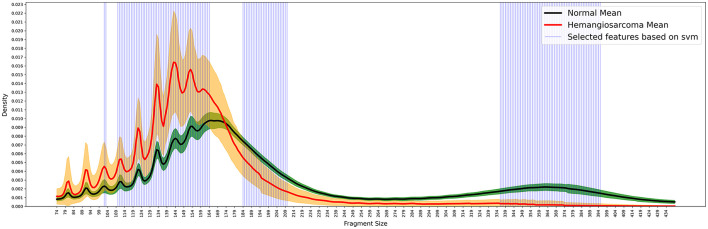
Selected featured based on SVM. We identified three key DNA regions from 100 iterative experiments using SVM. The blue lines indicate the important feature regions extracted using SVM. Fragment sizes were calculated in 5 bp increments, ranging from 74 to 434 bp. The *X*-axis represents fragment size, while the *Y*-axis indicates the proportion (density) of each size. In the graph, the red line represents the mean fragment size of hemangiosarcoma samples, with the orange color indicating the standard deviation. Similarly, the black line represents the mean fragment size of normal samples, and the green color denotes the standard deviation. We used the SVM feature selection method to extract the areas that showed the most distinct differences between the normal and cancer groups.

To delineate cancer-specific or notably significant genomic regions, our study leveraged the capabilities of machine learning through feature selection methodologies. By employing Support Vector Machine (SVM) models enhanced by Recursive Feature Elimination (RFE), we conducted a series of 100 iterative experiments. In these analyses, 70% of our dataset was allocated as training data, examining DNA fragment sizes spanning 74 base pairs (bp) to 439 bp. The identification of pivotal features resulted in their categorization into three distinct regions, as visually denoted by the blue line in Figure 3.

The inaugural region emerges at the third oscillation peak, capturing specific loci situated between the primary peaks observed in hemangiosarcoma and control samples. This delineation displays a pronounced variance in the distribution of short DNA fragments, underscoring the discrepancies highlighted in Section 3.1. The subsequent region, proximate to 174 bp, marks a juncture where the frequency of fragments begins to escalate in control samples. Conversely, the terminal region extends from 336 to 395 bp, where the discrepancy in fragment proportions between the two cohorts intensifies, only to abate beyond 395 bp.

Within these specified regions, the prevalence of fragments in control samples markedly surpasses that in hemangiosarcoma samples by a factor of at least 5–20-fold. This differential feature selection, facilitated by machine learning, underscores the utility of analyzing fragment size distributions in distinguishing between normal and hemangiosarcoma-affected canine DNA.

Subsequently, our study utilized machine learning algorithms to ascertain the presence of hemangiosarcoma within the samples. We employed a suite of seven classification algorithms across 10 repeated trials, with 70% of the data allocated for training purposes. The discriminative features incorporated into this phase were derived from the SVM model equipped with Recursive Feature Elimination (RFE), focusing on the peaks and valleys delineated in Section 3.1. As depicted in Table 3, the SVM approach consistently rendered commendable classification outcomes. Notably, the highest accuracy of 0.95 was achieved when the model utilized features representing peaks and valleys within human genomic areas, achieving an Area Under the Curve (AUC) of 0.93. Sensitivity was 0.89, but Specificity was notably high at 0.98, and the SVM algorithm showed the highest scores in all cases (Supplementary Table 4). A close second in performance was observed when incorporating features from canine-specific peaks, valleys, and the primary peak, culminating in an AUC of 0.92.

**Table 3:** Classification performance of hemangiosarcoma detection using machine learning algorithms and feature selection.

	**Pre-operative sample**			**Pre- and post-operative samples**		
	**SVM FS**	**Peaks, valleys and main peak of dog**	**Peaks and valleys of human** **(****14****)**	**SVM FS**	**Peaks, valleys and main peak of dog**	**Peaks and valleys of human**
ABC	0.9010	0.8667	0.8963	0.8516	0.8080	0.7821
BC	0.9123	0.8574	0.8929	0.8757	0.8240	0.8015
ETC	0.9070	0.9071	0.9025	0.8679	0.8386	0.8149
GBC	0.9082	0.8769	0.8577	0.8500	0.8260	0.7817
RF	0.9195	0.8714	0.8943	0.8836	0.8231	0.8205
SVM	0.8927	0.9204	0.9345	0.8996	0.8840	0.8820
XGBoost	0.8772	0.8571	0.8880	0.8759	0.8075	0.8017

The table values represent AUC, and Accuracy, Sensitivity, and Specificity are provided in Supplementary Table 4. FS, feature selection; ABC, AdaBoost Classifier; BC, Bagging Classifier; ETC, Extra Trees Classifier; GBC, Gradient Boosting Classifier; RF, Random Forest; SVM, Support Vector Machine; XGBoost, Extreme Gradient Boosting.

The analysis utilized 14 positions (comprising seven peaks and seven valleys) for human-related features and 21 positions (encompassing 10 peaks and 11 valleys) for canine-related features, underscoring the efficacy of achieving significant results with a concise set of features. Additional metrics beyond the AUC are accessible in Supplementary Table 4, providing a comprehensive overview of the classification performance.

Our investigation utilized data from the BioProject PRJNA823593, which comprises both pre-and post-operative samples from dogs diagnosed with hemangiosarcoma. We embarked on a classification task to differentiate between normal samples and those taken before and after surgery from affected dogs. The performance of these classifications, as quantified by the Area Under the Curve (AUC), is presented in Table 3. The analysis revealed a slightly lower AUC when attempting to distinguish pre-operative hemangiosarcoma samples from normal ones, compared to other classification tasks, with the most effective model being the Support Vector Machine (SVM) used for both feature selection and classification. This model achieved an AUC of 0.8996.

Leveraging machine learning techniques, we discovered specific genomic locations that exhibit significant differences between cancerous and non-cancerous states, including distinctive patterns in the DNA fragment size distribution, particularly in the peaks and valleys discussed in Section 3.1. The focal areas of interest spanned from the 5th to the 8th oscillation cycles, where notable discrepancies, including the principal peak, were observed. The positional patterns of peaks and valleys in both canine and human samples showed substantial alignment, with 11 identical positions noted. This congruency emphasizes the relevance and utility of a relatively small subset of features in achieving meaningful classification results.

Consequently, our findings underscore the efficacy of utilizing cell-free DNA (cfDNA) fragment size distribution as a potent marker for screening and diagnosing hemangiosarcoma in dogs. This demonstrates the method's potential applicability and precision in a clinical setting.

## 4 Discussion

Cancer remains a leading cause of mortality among canines, with hemangiosarcoma standing out as a notably aggressive form that predominantly affects organs like the liver, spleen, and heart. Often, by the time of its diagnosis through traditional tissue biopsy, the cancer has advanced significantly, underscoring the urgent need for earlier detection methods. In human oncology, considerable efforts are underway to identify reliable tumor markers for early diagnosis and prognosis, with cell-free DNA (cfDNA) emerging as a promising non-invasive diagnostic tool. This approach allows for cancer detection and monitoring through simple blood samples, circumventing the discomfort and risk associated with surgical biopsies. Drawing inspiration from human medical research, our study probes the utility of cfDNA as a potential tumor marker for canine cancer diagnostics. By analyzing the size distribution of cfDNA fragments through Whole Genome Sequencing (WGS) data, we aimed to discern distinct patterns between healthy and hemangiosarcoma-afflicted dogs.

Our investigation sought to determine if cell-free DNA (cfDNA) could be utilized as a biomarker for diagnosing, screening, and monitoring cancer in dogs, analogous to its application in human oncology. We conducted a pattern analysis focused on quantifying fragment sizes within cfDNA WGS data to achieve this. This analysis aimed to discern the variances in the distribution of fragment sizes between healthy canine samples and those afflicted with hemangiosarcoma, as depicted in Figure 2. Through this comparative approach, we aimed to identify distinct patterns that could potentially serve as indicators for the presence of hemangiosarcoma, thereby evaluating the feasibility of cfDNA as a non-invasive diagnostic tool in veterinary medicine.

Following the pattern analysis, we identified specific fragment sizes that exhibited notable differences between the control group and hemangiosarcoma-afflicted samples. Leveraging these distinct sizes, we employed machine learning techniques—specifically, Support Vector Machine (SVM)—to classify hemangiosarcoma. This approach was not limited to canine data; we also incorporated fragment sizes identified in human studies to enhance our classification model, thereby exploring the cross-species applicability of cfDNA fragment sizes as biomarkers for cancer detection. In addition to fragment size analysis, our study delved into detecting copy number alterations (CNAs) associated with hemangiosarcoma. To accomplish this, we utilized modified versions of the ichorCNA and WisecondorX algorithms tailored to our specific research needs. This comprehensive approach allowed us to identify and analyze CNAs that occur in hemangiosarcoma, contributing further to our understanding of the genetic underpinnings of this aggressive cancer in dogs. By integrating machine learning for fragment size classification and CNA analysis, our study aims to advance the diagnostic capabilities for hemangiosarcoma, potentially paving the way for early detection and more effective treatment strategies.

Our research aligns with existing studies on human cfDNA, demonstrating that the primary peak in healthy individuals typically measures around 167 base pairs (bp), contrasting with the shorter peak of ~163 bp observed in cancer patients. Similarly, our findings in canine subjects revealed a similar pattern: healthy dogs exhibited a main peak at 165 bp, whereas dogs with hemangiosarcoma showed a reduced peak length at 160 bp. This suggests a conserved mechanism influencing cfDNA fragmentation in both species in the context of cancer. Leveraging machine learning for data analysis, particularly employing the Support Vector Machine (SVM) model to focus on these significant peaks and valleys, yielded notable diagnostic accuracy. The model's performance was particularly effective when applying the SVM to the specific peaks and valleys characteristic of human cfDNA, achieving an AUC of 0.93, with sensitivity and specificity rates of 0.88 and 0.98, respectively. Utilizing canine-specific peaks, valleys, and main peak positions for classification also produced promising results, albeit slightly lower, with an AUC of 0.92, and sensitivity and specificity of 0.87 and 0.96.

Interestingly, including post-operative samples in our machine learning analysis diminished performance metrics, potentially reflecting a post-surgical reduction in disease burden rather than an improvement in the dogs' prognoses. This observation underscores the need for additional research incorporating a more extensive set of samples for monitoring purposes to interpret these findings more accurately. Reflecting on the broader implications of our study, the successful application of fragment size distribution and machine learning in the screening of hemangiosarcoma in dogs opens the door to the potential use of this methodology in other canine cancers. Given the ongoing research into cfDNA for cancer screening in humans, our results advocate for further exploration into the application of this non-invasive diagnostic tool across various cancer types in dogs, highlighting the value of cross-species insights in advancing cancer detection and treatment strategies.

In our exploration of the genetic landscape of canine hemangiosarcoma, we focused on identifying copy number alterations (CNAs) within genes previously associated with the disease, including CDKN2A/B, VEGFA, KDR, SKI, MYC, and KIT. A comprehensive CNA analysis identified genomic alteration patterns, revealing gains in 12 chromosomes and losses in 13 chromosomes. Notably, chromosomes 14 and 16 displayed both gains and losses, indicating significant overlapping events. However, further research is required to determine if these gains and losses are spatially separated within the same chromosome. Among the genes historically linked to hemangiosarcoma, alterations in CDKN2A/B, KDR, MYC, and KIT were prominently identified in our study, underscoring their potential roles in the pathogenesis of this cancer in dogs. The detection of KDR gains is particularly intriguing, given its documented involvement in human angiosarcoma, suggesting a possible conserved oncogenic pathway between species. However, our analysis also encountered limitations due to the sequencing depth, which hindered the accurate identification of specific mutations beyond CNAs that are known to be associated with hemangiosarcoma. This limitation highlights the necessity for further research employing more extensive WGS data. Notably, the work of Megquier et al. (37) illustrates the importance of such detailed genetic analysis, revealing significant CNA variations on chromosome 31 influenced by the presence or absence of PIK3CA mutations. This finding adds another layer of complexity to the genetic underpinnings of hemangiosarcoma. Our findings emphasize the need for more profound, more comprehensive genomic studies to fully elucidate the array of genetic mutations and CNAs driving hemangiosarcoma in dogs. Such research will enhance our understanding of the disease's molecular basis and pave the way for developing more effective diagnostic tools and therapeutic strategies, potentially benefiting both veterinary and comparative oncology.

Our study reveals that the patterns observed in canine cell-free DNA (cfDNA) closely mirror those seen in humans, marking a significant stride in the quest for reliable cancer biomarkers. Specifically, we identified discernible differences in the distribution of cfDNA fragment sizes between healthy dogs and those afflicted with hemangiosarcoma. These variances, illuminated through the lens of machine learning analysis, underscore the potential of cfDNA fragment size distribution as a viable tumor marker for screening and ongoing cancer monitoring in canines. In this study, significant discrepancies in sample sizes across stages, along with the limited overall sample size, prevented us from conducting an analysis on the impact and efficacy of the method at different stages of the disease. Because biomarkers can be increased with many states of disease and not only with cancer, the lack of comparison with inflammatory or non-neoplastic disease as a separate study group is an important limitation of this study. Increases in test results in these cases will decrease the specificity of this test. A future aim is to evaluate this test in the context of infectious, inflammatory, and other disease states as well as in other cancers. Additionally, although the CRP values for the normal samples were all within the normal range, indicating a very low likelihood of tumors or inflammatory conditions, further data or experiments beyond physical examination, serum chemistry, and CBC testing are needed to more rigorously validate the criteria for normal samples. This parallel between canine and human cfDNA patterns enhances our understanding of cancer's molecular underpinnings across species and solidifies the role of cfDNA analysis as a promising tool in the oncological arsenal. Given the burgeoning interest in cfDNA as a non-invasive diagnostic medium for various human cancers, our findings advocate for a broader application of this approach within veterinary medicine. There is a compelling need to extend cfDNA research to encompass a more comprehensive array of canine cancers, thereby harnessing its full potential for early detection, diagnosis, and monitoring, advancing both human and veterinary oncology.

In conclusion, this study demonstrates the potential of cell-free DNA (cfDNA) analysis as a non-invasive biomarker for the early detection and monitoring of canine hemangiosarcoma. By leveraging cfDNA fragment size distribution and copy number alteration (CNA) patterns, combined with machine learning models, we achieved high diagnostic accuracy, with an average Area Under the Curve (AUC) of 0.93. These findings align with similar patterns observed in human oncology, underscoring a possible cross-species conservation of cfDNA fragmentation in cancer. Furthermore, our CNA analysis identified significant genomic regions linked to hemangiosarcoma pathogenesis, such as alterations in CDKN2A/B, VEGFA, KDR, MYC, and KIT. Despite these promising results, the study has limitations, including the small sample size and the lack of a comprehensive assessment across different cancer stages. Future research should expand the sample size, investigate cfDNA patterns in other canine cancers, and refine machine learning models for broader clinical applicability. Overall, this study paves the way for integrating cfDNA-based liquid biopsy techniques into veterinary oncology, offering a minimally invasive and effective diagnostic tool for early cancer detection and personalized treatment strategies in dogs.

## Data Availability

The datasets presented in this study can be found in online repositories. The names of the repository/repositories and accession number(s) can be found below: https://www.ebi.ac.uk/ena, PRJNA823593.
