# Supplementary material for: Early detection of canine hemangiosarcoma via cfDNA fragmentation and copy number alterations in liquid biopsies using machine learning
Source: Front Vet Sci. 2025 Jan 13;11:1489402. doi: 10.3389/fvets.2024.1489402 (PMC11769935; doi:10.3389/fvets.2024.1489402)
Supplement: Supplementary file 1 [file Table_1.docx]

**Supplementary Table 1.** Clinical data information on hemangiosarcoma and normal (healthy) cases

| No | Patient | Gender | Breed | Age | BW(kg) | Diagnosis | cfDNA conc. |
| --- | --- | --- | --- | --- | --- | --- | --- |
| 1 | 16_oth | Ms | Standard Poodle | 14 | 25 | HSA I | 0.71 |
| 2 | 17_oth | Fs | Wheaten Terrier | 11 | 20 | HSA I | 4.77 |
| 3 | 19_oth | Fs | Labrador Retriever | 11 | 28 | HSA II | 0.22 |
| 4 | 20_oth | Ms | Labradoodle | 9 | 33 | HSA II | 10.47 |
| 5 | 21_oth | Fs | Labrador Retriever | 10 | 24 | HSA II | 4.26 |
| 6 | 22_oth | M | Goldendoodle | 12 | 56 | HSA II | 0.16 |
| 7 | 23_oth | Fs | German Shepherd | 11 | 36 | HSA II | 2.41 |
| 8 | 25_oth | M | Great Dane | 8 | 60 | HSA II | 1.23 |
| 9 | 26_oth | Ms | Mix | 11 | 32 | HSA II | 0.92 |
| 10 | 27_oth | Ms | Boxer mix | 6 | 37 | HSA II | 2.15 |
| 11 | 28_oth | Fs | German Shepherd | 12 | 38 | HSA II | 35.93 |
| 12 | 29_oth | Fs | Boxer | 7 | 28 | HSA II | 175.8 |
| 13 | 30_oth | Ms | English Setter | 11 | 26 | HSA III | 0.08 |
| 14 | 33_oth | Ms | Yorkshire Terrier Mix | 8 | 7 | HSA III | 21.25 |
| 15 | 34_oth | Fs | Mix | 9 | 18 | HSA III | 0.6 |
| 16 | 35_oth | Fs | Mix | 11 | 21 | HSA III | 9.31 |
| 17 | 36_oth | Fs | Mix | 12 | 38 | HSA III | 0.85 |
| 18 | 37_oth | Ms | Husky | 10 | 34 | HSA III | 87.74 |
| 19 | 38_oth | M | Golden Retriever | 11 | 35 | HSA III | 28.43 |
| 20 | 13_oth | Fs | Corgi | 14 | 11 | Sarcoma | 15.75 |
| 21 | 39_oth | Fs | Mix | 12 | 22 | Sarcoma | 23.57 |
| 22 | 2_oth | Ms | American Pit Bull | 6 | 40 | normal | 4.38 |
| 23 | 3_oth | Ms | Mix | 10 | 25 | normal | 1.06 |
| 24 | 5_oth | Ms | Mix | 12 | 35 | normal | 2.2 |
| 25 | 6_oth | Fs | Labrador Retriever | 6 | 31 | normal | 1.91 |
| 26 | 7_oth | Ms | Pointer mix | 9 | 39 | normal | 5.02 |
| 27 | 8_oth | Fs | American Pit Bull | 7 | 39 | normal | 0.39 |
| 29 | 10_oth | Fs | English Bulldog | 10 | 25 | normal | 0.28 |
| 31 | 12_oth | Ms | Jack Russel Terrier | 13 | 10 | normal | 0.54 |
| 32 | 15_oth | Fs | Labrador Retriever | 11 | 63 | normal | 0.17 |
| 33 | 1_our | Fs | Border Collie | 2 | 15 | normal | 0.94 |
| 34 | 2_our | M | Border Collie | 3 | 17 | normal | 0.92 |
| 35 | 3_our | Fs | Border Collie | 13 | 17 | normal | 2.61 |
| 36 | 4_our | Fs | Border Collie | 3 | 18 | normal | 1.83 |
| 37 | 5_our | Fs | Border Collie | 13 | 17 | normal | 1.19 |
| 38 | 6_our | Ms | Border Collie | 13 | 21 | normal | 1.82 |
| 39 | 7_our | Fs | Border Collie | 9 | 16 | normal | 2.33 |
| 40 | 8_our | Ms | Border Collie | 9 | 22 | normal | 4.09 |
| 41 | 9_our | Ms | Border Collie | 7 | 21 | normal | 1.41 |
| 42 | 10_our | Fs | Border Collie | 7 | 17 | normal | 1.79 |
| 43 | 11_our | Ms | Border Collie | 7 | 22 | normal | 1.30 |
| 44 | 12_our | Ms | Border Collie | 6 | 20 | normal | 2.40 |
| 45 | 13_our | Ms | Labrador Retriever | 13 | 33 | normal | 3.00 |
| 46 | 14_our | Fs | Golden Retriever | 11 | 25 | normal | 1.36 |
| 47 | 15_our | Fs | Golden Retriever | 13 | 23 | normal | 3.01 |
| 48 | 16_our | Fs | Labrador Retriever | 11 | 25 | normal | 1.19 |
| 49 | 17_our | Fs | Labrador Retriever | 13 | 26 | normal | 2.16 |
| 50 | 18_our | Fs | Labrador Retriever | 11 | 25 | normal | 1.30 |
| 51 | 19_our | Fs | Labrador Retriever | 11 | 26 | normal | 1.72 |
| 52 | 20_our | Ms | Labrador Retriever | 10 | 34 | normal | 1.48 |
| 53 | 21_our | Fs | Labrador Retriever | 7 | 24 | normal | 2.80 |
| 54 | 22_our | Ms | Labrador Retriever | 7 | 33 | normal | 2.48 |
| 55 | 23_our | Ms | Labrador Retriever | 7 | 35 | normal | 2.81 |
| 56 | 24_our | Fs | Labrador Retriever | 7 | 27 | normal | 4.69 |
| 57 | 25_our | Fs | Labrador Retriever | 5 | 27 | normal | 1.71 |
| 58 | 26_our | Fs | Labrador Retriever | 3 | 23 | normal | 1.09 |
| 59 | 27_our | Ms | Labrador Retriever | 4 | 28 | normal | 3.09 |
