# Supplementary material for: Early detection of canine hemangiosarcoma via cfDNA fragmentation and copy number alterations in liquid biopsies using machine learning
Source: Front Vet Sci. 2025 Jan 13;11:1489402. doi: 10.3389/fvets.2024.1489402 (PMC11769935; doi:10.3389/fvets.2024.1489402)
Supplement: Supplementary file 2 [file Table_2.docx]

**Supplementary Table 2.** Clinical data information of normal (healthy) cases

All client-owned dogs were determined to be normal through physical examination and underwent CRP, serum chemistry, and CBC testing. As all CRP levels were within the normal range, the likelihood of tumors, inflammatory conditions, or other non-neoplastic diseases was considered very low. The results of serum chemistry and CBC were largely within normal ranges, with values exceeding the normal range highlighted in gray. Furthermore, no evidence of tumors or inflammatory conditions was observed within one year following blood sample collection.

1. Results of serum chemistry

| Name | Glucose | Creatinine | BUN | BUN : Creatinine Ratio | Phosphorus-Inorganic | Calcium[Ca++] | Protein-Total | Albumin | Globulin | Albumin : Globulin Ration | Alanine Aminotransferase | Alkaline Phosphatase | Gamma Glutamyltransferase | Bilirubin-Total | Cholesterol-Total | Amylase | Lipase | CRP |
| --- | --- | --- | --- | --- | --- | --- | --- | --- | --- | --- | --- | --- | --- | --- | --- | --- | --- | --- |
| Unit | mg/dL | mg/dL | mg/dL |  | mg/dL | mg/dL | g/dL | g/dL | g/dL |  | U/L | U/L | U/L | mg/dL | mg/dL | U/L | U/L |  |
| Min | 70 | 0.5 | 7 |  | 2.5 | 7.9 | 5.2 | 2.2 | 2.5 |  | 10 | 23 | 0 | 0 | 110 | 500 | 200 | 10 |
| Max | 143 | 1.8 | 27 |  | 6.8 | 12 | 8.2 | 3.9 | 4.5 |  | 125 | 212 | 11 | 0.9 | 320 | 1500 | 1800 | 20 |
| 1_our | N | N | N | N | N | N | N | N | N | N | N | N | N | N | N | N | N | N |
| 2_our | N | N | N | N | N | N | N | N | N | N | N | N | N | N | N | 421 | N | N |
| 3_our | N | N | N | N | N | N | N | N | N | N | N | N | N | N | N | N | N | N |
| 4_our | N | N | N | N | N | N | N | N | N | N | N | N | N | N | N | N | N | N |
| 5_our | N | N | N | N | N | N | N | N | N | N | N | N | N | N | N | N | N | N |
| 6_our | N | N | N | N | N | N | N | N | N | N | N | N | N | N | N | N | N | N |
| 7_our | N | N | N | N | N | N | 8.5 | N | N | N | N | N | N | N | N | N | N | N |
| 8_our | N | N | N | N | N | N | N | N | N | N | N | N | N | N | N | N | N | N |
| 9_our | N | N | N | N | N | N | N | N | N | N | N | N | N | N | N | N | N | N |
| 10_our | N | N | N | N | N | N | N | N | N | N | N | N | N | N | N | N | N | N |
| 11_our | N | N | N | N | N | N | N | N | N | N | N | N | N | N | N | N | N | N |
| 12_our | N | N | N | N | N | N | N | N | N | N | N | N | N | N | N | N | N | N |
| 13_our | N | N | N | N | N | N | N | N | N | N | N | N | N | N | N | N | N | N |
| 14_our | N | N | N | N | N | N | N | N | N | N | N | N | N | N | N | N | N | N |
| 15_our | N | N | N | N | N | N | N | 4.0 | N | N | N | N | N | N | N | N | N | N |
| 16_our | N | N | N | N | N | N | N | N | N | N | N | N | N | N | N | N | N | N |
| 17_our | N | N | N | N | N | N | N | N | N | N | N | N | N | N | N | N | N | N |
| 18_our | N | N | N | N | N | N | N | N | N | N | N | N | N | N | N | N | N | N |
| 19_our | N | N | N | N | N | N | N | N | N | N | N | N | N | N | N | N | N | N |
| 20_our | N | 1.9 | N | N | N | N | N | N | N | N | N | N | N | N | N | N | N | N |
| 21_our | N | N | N | N | N | N | N | N | N | N | N | N | N | N | N | N | N | N |
| 22_our | N | N | N | N | N | N | N | N | N | N | N | N | N | N | N | N | N | N |
| 23_our | N | N | N | N | N | N | N | N | N | N | N | N | N | N | N | N | N | N |
| 24_our | N | N | N | N | N | 13.0 | N | N | N | N | N | N | N | N | N | N | N | N |
| 25_our | N | N | N | N | N | N | N | N | N | N | N | N | N | N | N | N | N | N |
| 26_our | N | N | N | N | 7.0 | N | N | N | N | N | N | N | N | N | N | N | N | N |
| 27_our | N | N | N | N | N | N | N | N | N | N | N | N | N | N | N | N | N | N |

1. CBC(Complete Blood Count) results

|  | RBC | HGB | MCV | MCH | MCHC | RDW | RETIC | RETIC-HGB | WBC | NEU | LYM | MONO | EOS | BASO | PLT | MPV | PDW | PCT |
| --- | --- | --- | --- | --- | --- | --- | --- | --- | --- | --- | --- | --- | --- | --- | --- | --- | --- | --- |
| Unit | 10x12/L | g/dL | fL | pg | g/dL | % | K/μL | pg | 10x9/L | K/μL | K/μL | K/μL | K/μL | K/μL | K/μL | fL | fL | % |
| Min | 5.65 | 13.1 | 61.6 | 21.2 | 32 | 13.6 | 10 | 22.3 | 5.05 | 2.95 | 1.05 | 0.16 | 0.06 | 0 | 148 | 8.7 | 9.1 | 0.14 |
| Max | 8.87 | 20.5 | 73.5 | 25.9 | 37.9 | 21.7 | 110 | 29.6 | 16.76 | 11.64 | 5.1 | 1.12 | 1.23 | 0.1 | 484 | 13.2 | 19.4 | 0.46 |
| 1_our | N | N | N | 18.9 | N | N | N | N | N | N | N | N | N | N | N | N | N | N |
| 2_our | N | N | N | N | N | N | N | N | N | N | N | N | N | N | N | N | N | N |
| 3_our | N | N | N | N | N | N | N | N | N | N | N | N | N | N | N | N | N | N |
| 4_our | N | N | 73.6 | N | N | N | N | N | N | N | N | N | N | N | N | N | N | N |
| 5_our | N | 20.8 | N | N | N | N | N | N | N | N | N | N | N | N | N | N | N | N |
| 6_our | N | N | N | N | N | N | N | N | N | N | N | N | N | N | N | N | N | N |
| 7_our | N | N | N | N | N | N | N | N | N | N | N | N | N | N | N | N | N | N |
| 8_our | N | N | N | N | N | N | N | N | N | N | N | N | N | N | N | 13.9 | N | N |
| 9_our | N | N | N | N | N | N | N | N | N | N | N | N | N | N | N | N | N | N |
| 10_our | N | N | N | N | N | N | N | N | N | N | N | N | N | N | N | N | N | N |
| 11_our | N | N | N | N | N | N | N | N | N | N | N | N | N | N | N | 13.4 | N | N |
| 12_our | N | N | N | N | N | N | N | N | N | N | 5.70 | N | N | N | N | N | N | N |
| 13_our | N | N | N | N | N | N | N | N | N | N | N | N | N | N | N | N | N | N |
| 14_our | N | N | N | N | N | N | N | N | N | N | N | N | N | N | N | N | N | N |
| 15_our | N | N | N | N | N | N | N | N | N | N | N | N | N | N | N | 13.9 | N | N |
| 16_our | N | N | N | N | N | N | N | N | N | N | N | N | N | N | N | N | N | N |
| 17_our | N | N | N | N | N | N | N | N | N | N | N | N | N | N | N | 13.3 | N | N |
| 18_our | N | N | N | N | N | N | N | N | N | N | 5.54 | N | N | N | N | N | N | N |
| 19_our | N | N | N | N | N | N | N | N | N | 12.00 | N | N | N | N | N | N | N | N |
| 20_our | N | N | N | N | N | N | N | N | N | N | 5.13 | N | N | N | N | N | N | N |
| 21_our | N | N | N | N | N | N | N | N | N | N | N | N | N | N | N | 13.3 | N | N |
| 22_our | N | N | N | N | N | N | N | N | N | N | N | N | N | N | N | N | N | N |
| 23_our | N | N | N | N | N | N | N | N | N | N | N | N | N | N | N | N | N | N |
| 24_our | N | N | N | N | N | N | N | N | N | N | N | N | N | N | N | N | N | N |
| 25_our | N | N | N | N | N | N | N | N | N | N | 5.19 | N | N | N | N | N | N | N |
| 26_our | N | N | N | N | N | N | N | N | N | N | N | 1.14 | N | N | N | N | N | N |
| 27_our | N | N | N | N | N | N | N | N | N | N | N | 1.23 | N | N | N | N | N | N |

The "N" in the cell indicates values within the normal range and stands for "Normal”.
