# Supplementary material for: Early detection of canine hemangiosarcoma via cfDNA fragmentation and copy number alterations in liquid biopsies using machine learning
Source: Front Vet Sci. 2025 Jan 13;11:1489402. doi: 10.3389/fvets.2024.1489402 (PMC11769935; doi:10.3389/fvets.2024.1489402)
Supplement: Supplementary file 3 [file Table_3.docx]

**Supplementary Table 3.** The gain and loss region of hemangiosarcoma

| Gain | | | Loss | | |
| --- | --- | --- | --- | --- | --- |
| Chr | Start | End | Chr | Start | End |
| 5 | 500001 | 89000000 | 2 | 6500001 | 85500000 |
| 6 | 1000001 | 45000000 | 10 | 500001 | 69000000 |
| 13 | 500001 | 63000000 | 11 | 1500001 | 74500000 |
| 14 | 3000001 | 61000000 | 14 | 3000001 | 61000000 |
| 16 | 500001 | 59000000 | 16 | 500001 | 59000000 |
| 20 | 500001 | 57000000 | 27 | 500001 | 45500000 |
| 24 | 1000001 | 47000000 | 30 | 1000001 | 40000000 |
| 31 | 500001 | 39500000 | 33 | 1 | 31500000 |
|  |  |  | 34 | 500001 | 42000000 |
|  |  |  | 36 | 1 | 31000000 |
