# Supplementary material for: Early detection of canine hemangiosarcoma via cfDNA fragmentation and copy number alterations in liquid biopsies using machine learning
Source: Front Vet Sci. 2025 Jan 13;11:1489402. doi: 10.3389/fvets.2024.1489402 (PMC11769935; doi:10.3389/fvets.2024.1489402)
Supplement: Supplementary file 4 [file Table_4.docx]

**Supplementary Table 4.** Machine learning Results Summary for Hemangiosarcom and Normal

|  |  | Pre-operative sample | | | | Pre- and post-operative samples | | | |
| --- | --- | --- | --- | --- | --- | --- | --- | --- | --- |
| Method of feature selection |  | AUC | Accuracy | Sensitivity | Specificity | AUC | Accuracy | Sensitivity | Specificity |
| SVMFS | ABC | 0.9010 | 0.9158 | 0.8528 | 0.9492 | 0.8516 | 0.8452 | 0.8342 | 0.8690 |
|  | BC | 0.9123 | 0.9316 | 0.8504 | 0.9742 | 0.8757 | 0.8742 | 0.8851 | 0.8663 |
|  | ETC | 0.9070 | 0.9158 | 0.8814 | 0.9326 | 0.8679 | 0.8742 | 0.9222 | 0.8135 |
|  | GBC | 0.9082 | 0.9316 | 0.8338 | 0.9826 | 0.8500 | 0.8516 | 0.8536 | 0.8463 |
|  | RF | 0.9195 | 0.9368 | 0.8647 | 0.9742 | 0.8836 | 0.8903 | 0.9283 | 0.8390 |
|  | SVM | 0.8927 | 0.9211 | 0.8028 | 0.9826 | 0.8996 | 0.8839 | 0.8301 | 0.9691 |
|  | XGBoost | 0.8772 | 0.9053 | 0.7885 | 0.9659 | 0.8759 | 0.8806 | 0.9075 | 0.8444 |
| Peaks,Valleys and main peak of dog | ABC | 0.8667 | 0.8842 | 0.7937 | 0.9398 | 0.8080 | 0.8226 | 0.8531 | 0.7629 |
|  | BC | 0.8574 | 0.8737 | 0.7812 | 0.9337 | 0.8240 | 0.8355 | 0.8629 | 0.7852 |
|  | ETC | 0.9071 | 0.9158 | 0.8635 | 0.9507 | 0.8386 | 0.8484 | 0.8809 | 0.7963 |
|  | GBC | 0.8769 | 0.8895 | 0.8117 | 0.9420 | 0.8260 | 0.8290 | 0.8553 | 0.7967 |
|  | RF | 0.8714 | 0.8895 | 0.7937 | 0.9491 | 0.8231 | 0.8290 | 0.8575 | 0.7886 |
|  | SVM | 0.9204 | 0.9316 | 0.8746 | 0.9663 | 0.8840 | 0.8677 | 0.8224 | 0.9455 |
|  | XGBoost | 0.8571 | 0.8737 | 0.7825 | 0.9317 | 0.8075 | 0.8161 | 0.8521 | 0.7629 |
| Peaks and Valleys of human | ABC | 0.8963 | 0.9105 | 0.8354 | 0.9573 | 0.7821 | 0.7903 | 0.8355 | 0.7287 |
|  | BC | 0.8929 | 0.9105 | 0.8279 | 0.9580 | 0.8015 | 0.8097 | 0.8505 | 0.7525 |
|  | ETC | 0.9025 | 0.9158 | 0.8671 | 0.9379 | 0.8149 | 0.8194 | 0.8453 | 0.7846 |
|  | GBC | 0.8577 | 0.8737 | 0.8029 | 0.9126 | 0.7817 | 0.7903 | 0.8345 | 0.7290 |
|  | RF | 0.8943 | 0.9105 | 0.8404 | 0.9482 | 0.8205 | 0.8226 | 0.8397 | 0.8014 |
|  | SVM | 0.9345 | 0.9474 | 0.8871 | 0.9818 | 0.8820 | 0.8677 | 0.8128 | 0.9512 |
|  | XGBoost | 0.8880 | 0.9053 | 0.8279 | 0.9482 | 0.8017 | 0.8065 | 0.8334 | 0.7701 |
