# Supplementary material for: Early detection of canine hemangiosarcoma via cfDNA fragmentation and copy number alterations in liquid biopsies using machine learning
Source: Front Vet Sci. 2025 Jan 13;11:1489402. doi: 10.3389/fvets.2024.1489402 (PMC11769935; doi:10.3389/fvets.2024.1489402)
Supplement: Supplementary file 5 [file Table_5.docx]

**Supplementary Table 5.** The gain and loss region of hemangiosarcoma

| Gain KEGG pathway | | | | | |
| --- | --- | --- | --- | --- | --- |
| Term | % | Pvalue | Bonferroni | Benjamini | FDR |
| cfa04670:Leukocyte transendothelial migration | 1.027146001 | 1.55E-05 | 0.005364499 | 0.005378898 | 0.004960367 |
| cfa04062:Chemokine signaling pathway | 1.369528002 | 8.95E-05 | 0.030566456 | 0.015178193 | 0.013997181 |
| cfa04530:Tight junction | 1.320616288 | 1.31E-04 | 0.044516293 | 0.015178193 | 0.013997181 |
| cfa05167:Kaposi sarcoma-associated herpesvirus infection | 1.345072145 | 4.97E-04 | 0.15837193 | 0.034613574 | 0.031920299 |
| cfa04015:Rap1 signaling pathway | 1.491807288 | 4.99E-04 | 0.158955782 | 0.034613574 | 0.031920299 |
| cfa04727:GABAergic synapse | 0.758131573 | 9.82E-04 | 0.288884262 | 0.05679211 | 0.052373128 |
| cfa04724:Glutamatergic synapse | 0.880410858 | 0.001321383 | 0.367973029 | 0.059517136 | 0.05488612 |
| cfa04935:Growth hormone synthesis, secretion and action | 0.880410858 | 0.001564595 | 0.419194699 | 0.059517136 | 0.05488612 |
| cfa04926:Relaxin signaling pathway | 0.95377843 | 0.001674446 | 0.440951881 | 0.059517136 | 0.05488612 |
| cfa04713:Circadian entrainment | 0.78258743 | 0.001715191 | 0.448813673 | 0.059517136 | 0.05488612 |
| cfa04725:Cholinergic synapse | 0.855955001 | 0.002239965 | 0.540740356 | 0.067286939 | 0.062051356 |
| cfa04514:Cell adhesion molecules | 1.027146001 | 0.002326926 | 0.554422533 | 0.067286939 | 0.062051356 |
| cfa04390:Hippo signaling pathway | 1.12496943 | 0.002547811 | 0.587376095 | 0.068006968 | 0.062715359 |
| cfa04921:Oxytocin signaling pathway | 1.051601859 | 0.003356354 | 0.688580235 | 0.080244405 | 0.074000604 |
| cfa05160:Hepatitis C | 1.100513573 | 0.003468778 | 0.700535243 | 0.080244405 | 0.074000604 |
| cfa05032:Morphine addiction | 0.709219858 | 0.003707421 | 0.724416784 | 0.080404686 | 0.074148414 |
| cfa04726:Serotonergic synapse | 0.831499144 | 0.004340064 | 0.778930878 | 0.083722199 | 0.077207791 |
| cfa05163:Human cytomegalovirus infection | 1.418439716 | 0.004342938 | 0.779152196 | 0.083722199 | 0.077207791 |
| cfa05135:Yersinia infection | 0.929322573 | 0.004861758 | 0.815691508 | 0.084635069 | 0.078049631 |
| cfa04360:Axon guidance | 1.222792859 | 0.004878102 | 0.816738912 | 0.084635069 | 0.078049631 |
|  | | | | | |
| Loss KEGG pathway | | | | | |
| Term | % | Pvalue | Bonferroni | Benjamini | FDR |
| cfa04742:Taste transduction | 0.987456632 | 4.97E-09 | 1.73E-06 | 1.73E-06 | 1.54E-06 |
| cfa04060:Cytokine-cytokine receptor interaction | 1.974913264 | 6.21E-05 | 0.021443446 | 0.010692507 | 0.009497642 |
| cfa05163:Human cytomegalovirus infection | 1.627969042 | 1.19E-04 | 0.040787747 | 0.010692507 | 0.009497642 |
| cfa04934:Cushing syndrome | 1.227648786 | 1.23E-04 | 0.041870801 | 0.010692507 | 0.009497642 |
| cfa04550:Signaling pathways regulating pluripotency of stem cells | 1.120896717 | 4.30E-04 | 0.139428548 | 0.025342089 | 0.022510165 |
| cfa05167:Kaposi sarcoma-associated herpesvirus infection | 1.387776888 | 4.85E-04 | 0.155627147 | 0.025342089 | 0.022510165 |
| cfa04630:JAK-STAT signaling pathway | 1.227648786 | 5.08E-04 | 0.162588525 | 0.025342089 | 0.022510165 |
| cfa04820:Cytoskeleton in muscle cells | 1.627969042 | 7.96E-04 | 0.242650549 | 0.034727484 | 0.030846762 |
| cfa04936:Alcoholic liver disease | 1.040832666 | 9.98E-04 | 0.294323783 | 0.038713858 | 0.034387668 |
| cfa04928:Parathyroid hormone synthesis, secretion and action | 0.907392581 | 0.001431173 | 0.393370543 | 0.046066252 | 0.040918447 |
| cfa05165:Human papillomavirus infection | 2.108353349 | 0.001451945 | 0.397758651 | 0.046066252 | 0.040918447 |
| cfa00052:Galactose metabolism | 0.346944222 | 0.002022643 | 0.506689943 | 0.058825211 | 0.05225162 |
| cfa04270:Vascular smooth muscle contraction | 1.014144649 | 0.002247775 | 0.544042829 | 0.060344109 | 0.053600784 |
| cfa05166:Human T-cell leukemia virus 1 infection | 1.467840939 | 0.002882851 | 0.634894224 | 0.071865367 | 0.063834567 |
| cfa05170:Human immunodeficiency virus 1 infection | 1.414464905 | 0.003424685 | 0.69798132 | 0.078304169 | 0.069553846 |
| cfa04350:TGF-beta signaling pathway | 0.854016547 | 0.003608805 | 0.716842186 | 0.078304169 | 0.069553846 |
| cfa04912:GnRH signaling pathway | 0.720576461 | 0.003814243 | 0.736503726 | 0.078304169 | 0.069553846 |
| cfa00514:Other types of O-glycan biosynthesis | 0.45369629 | 0.004258821 | 0.77451519 | 0.082573804 | 0.073346359 |
| cfa04916:Melanogenesis | 0.773952495 | 0.004545229 | 0.796054191 | 0.083488683 | 0.074159002 |
|  |  |  |  |  |  |
